# Report of Four Cases of Enterotomy

**Published:** 1888-06

**Authors:** Edward Von Donhoff

**Affiliations:** Athens, Ga.; Formerly of Louisville, Ky.


### REPORT OF FOUR CASES OF ENTEROTOMY.

BY EDWARD VON DONHOFF, M. S., M. D., ATHENS, GA.
(Formerly of Louisville, Ky.)

The following cases will prove of interest and, it is to be hoped, point a useful lesson. The author is identified with the national method in surgery (vide, Ky. State Med. Society, meeting 1886.), and as proven by the results detailed here, sees no reason to unsay any part of his position.

CASE I—N. L., white, age thirty-five, laborer, admitted to the City Hospital, Louisville, Ky., July, 1878.

Twenty-eight hours before admission, the patient, an employe of the L. & N. R. R., had sustained a strangulation of an old hernial protrusion, in the right inguinal region.

*Present Condition*—Patient semi-conscious, pulse full, corded and rapid, temperature 105°, abdomen intensely tympanitic, features extremely pinched and anxious, perspiration labored and shallow, occasional stercoracious vomiting, and suppression of urine.

In the right inguinal region, at a point corresponding to the internal ring, a tumor of conical shape, and the size of a turkey-egg, extremely tense (almost to inelasticity), presented.

*Diagnosis*—Strangulated inguinal hernia.

*Treatment*—Four hours after admission, when I had reached the hospital, herniotomy was done, and the strangulated gut relieved; the sac was cut away, its cut edges brought together and the abdominal ring closed with sutures, which were made to include all superjacent structures.

Two hours afterward the patient having rallied from the anæsthesia, vomiting returned, and the usual symptoms of existing inter-abdominal strangulation presented. I decided upon adding laparotomy to the already protracted interference. The abdomen was opened. The omentum was found infiltrated, and covered with lymph-coagula, and lightly adherent to the underlying coils of inflated intestines, which were, in part, matted together An intussusception of fourteen inches in length was found in the lower portion, near the ileo-cœcal valve of the ilium. On being gently relieved the invaginated portion of gut was found sphacelated; this was excised. The messentery was caught up in loops and tied with organic ligature. The proximal end was then invaginated—by means of a cylinder of stearine previously put into it—for a distance of two inches into the distal segment. The operation being completed with a modified Lambert suture. The cylinder of stearine was then traced onward until it passed the valve. The abdominal cavity, having been thoroughly irrigated with a weak, warm solution of salt, in water, was closed with closely arranged points of penetrating and superficial interrupted sutures. The usual external dressings were applied. A "hypodermic" of half grain morphiæ sulph. was administered and repeated in five hours. With little interruption the patient made a good recovery at the end of five weeks in hospital.

CASE II.—Negro man, admitted to United States Marine Hospital, Louisville, Ky. By the courtesy of Dr. Jno. Godfrey, surgeon in charge, I saw, and with his masterly assistance, operated on the case.

*Previous History*—Forty-eight hours before admission, the patient, a negro, robust, of tall stature, light brown complexion, and medium weight, sustained a strangulation of an old right inguino scrotal hernia.

*Present Condition*—Patient has extremely anxious appearance, small quick pulse, shallow respiration, temperature 102°, suppression of urine. and slight vomiting. At the right inguino-scrotal region is a very large tumor, which is plainly of a fluctuating character, and has the gross appearance of hydrocele—having an inverted pyriform shape, and being partly translucent. With an aspirator a portion of the contained slightly bloody serous fluid was withdrawn.

*Diagnosis*—Strangulated hernia—the original hernia probably cogenital—with hydrocele. [The patient's intelligence was not equal to the task of offering explanations.]

*Treatment*—The cut made traversed, transversely, the neck of the tumor and extended, about four inches, into the dartos. Rapid dissection discovered the sac to be composed of the tunica vaginalis, *i. e.*, externally (?). This was carefully scraped open, when a gush of serum verified a part of the diagnosis. The opening being extended, revealed a coil of ilium—sphacilated—lying in the upper portion of the sac in close contact with the spermatic cord. The strangulation was relieved with the herniatome. The intestine was then gently drawn downward and inspected. A section nine inches in length, was removed in manner detailed above, and the divided ends treated in a similar fashion—sac excised and wound closed as before described. Patient in extreme shock, temperature 96°; survived fourteen hours. Previous to death vomiting had ceased and patient had one alvine discharge.

*Autopsy*—Gut-suture intact; cylinder of stearine disappeared; evidence of intense general peritonitis marked; heart dilated and fatty.

CASE III.—Mrs. K., patient of my father, Dr. Alb. von Donhoff, æt., sixty-four; subject of femoral hernia.

*Present Condition*—Ten hours after symptoms of strangulation of the gut, a tumor, tense and dark-purple in appearance, and as large as one's closed hand, presents in the right femoral region, vomiting, auria, swollen, tense and painful belly and colliquative sweat observable, features pale and anxious, temperature 98°, pulse feeble, 120 per minute.

*Diagnosis*—Strangulated femoral hernia.

*Treatment*—A lifted, curvilinear flap of skin and superficial fascia, revealed the hernia tumor lying outside the cribriform fascia, in the upper portion of Scarpo's triangle. The sac contained a mass of small intestine and the caput coli enveloped in omentum, the latter being intensely infiltrated and bound together with strong fibrous bands. The small intestine in the sac and the appendix vermiformis were caught between strongly constricting bands.

and were gangrenous. A tedious dissection relieved the parts involved in the mass. The appendix vermiformis was ligatured close to colon and cut off. The small gut involved was excised (seventeen inches) and treated as above. Sac partly excised. External wound closed with interrupted suture.

Patient recovered after a severe illness, lasting five weeks. The greatest part of the external wound healed by granulation.

CASE IV.—Mandy McK., servant woman, æt. thirty-five, patient of Dr. T. H. Stucky, Louisville, Kentucky, and at the time of present seizure (January, 1887) convalescent of typhoid fever. On the day I was called she had risen from her bed, and in attempting to cross the floor, caught her foot in a rent in the carpet and was thrown, violently, on her back and side. Dr. S. saw patient a short time after the accident and pronounced sub-normal temperature, small pulse, quick, shallow respiration, vomiting and extreme facial anxiety. Bladder contained small quantity of dark urine. Great pain in the belly, especially about umbilicus.

*Present Condition*—Eight hours after accident I saw the woman with Drs. S. and Mills, and found her in impending syncope. Abdomen distended and very sensitive. Bladder empty, pulse feeble, temperature 97.

*Diagnosis*—Volvulus and, probably, rupture of mesenterium and ilium.

*Treatment*—Anæsthesia induced with ether after the patient had received 1.75 grain of atropia sulph. hypoderm. and a small quantity of diluted whiskey per orem. The abdomen was rapidly opened and revealed the cavity charged with large masses of blood-clots, amounting, apparently, to three or four pounds. These were turned out; the cavity was thoroughly irrigated with a tepid weak solution of chloride sod., after which, the cavity being examined, a large rent of the mesenterium was discovered opposite a point in the ilium, situate about twenty inches from the "valve." The ilium was also perforated at this point. The injured parts were treated as nearly secundem artem as possible. The patient revived and survived the operation, having meanwhile partaken of some nourishment and drink, which she retained, until the lapse of thirty hours when she expired suddenly. [A severe pneumonitis. in my own person, immediately followed my engagement with this case and so relegated it to Dr. S. during its subsequent continuance.]

Comments are, seemingly, unnecessary. These cases constitute a portion of my experience in laparotomy, the greater part of which, gathered without the aid (?) of chemical antiseptics (?), has been gratifying. I invariably require the apartment in which such an operation is to be done to be heated to 100° Farenheit at least.

